# A Course of Clinical Lectures, Delivered at the Hôtel Dieu, Paris, for the Session 1842-’43

**Published:** 1843-05-27

**Authors:** A. F. Chomel


THE MEDICAL EXAMINER,

And Retrospect of the Medical Sciences.

Vol. VI.] PHILADELPHIA, SATURDAY, MAY 27, 1843. [No. 10.

### A COURSE OF CLINICAL LECTURES,

*Delivered at the Hôtel Dieu, Paris, for the Session* 1842–'43.

BY A. F. CHOMEL, M. D.

### LECTURE VIII.

### ILIAC ABSCESS.

I shall to-day, commenced Dr. Chomel, direct your attention to the interesting subject of those purulent collections which occur in the iliac region. Three cases of this kind have lately presented themselves to our notice in these wards. One terminated fatally last month; and the other two are still in the hospital.

CASE I.—*Post puerperal peritonitis—Consecutive iliac abscess—Spontaneous perforation—Acute peritonitis —Death.*

In the course of last month you may remember a patient at No. 29 of the Salle Saint-Bernard. This woman entered the house with symptoms of acute peritonitis. She had been recently delivered, and imprudently left the *Maternity* three days after her confinement. She was almost immediately seized with pain in the abdomen, with general malaise, and fever. When she was admitted, on the 17th of December, she suffered a great deal from pain in the hypogastric region; pressure there was very painful; and the uterus was felt quite voluminous. There was want of appetite, with fever; there was, in a word, post-puerperal peritonitis, an affection much less grave, as you know, than puerperal peritonitis, properly so called. This affection was combatted by general bleedings, by the application of leeches on the seat of pain, by poultices, and by revulsives to the intestines. Under the influence of this treatment, the general and local condition was ameliorated; but at the same time an acute pain was developed in the right iliac region; then an elastic tumor appeared, which was distinctly felt by pressing firmly on this region with the fingers. The abdominal parietes were stretched, and could be depressed with difficulty; the sensibility was very much exalted, and the fever reappeared. By these symptoms it was not difficult to recognise a phlegmonous tumor situated in the cellular tissue of the iliac fossa.

These kinds of tumors increase towards the pelvis sometimes, and point towards the external organs of generation, particularly in the vagina. Hence, on carrying the finger into this canal, and pressing on the prominence of the tumor, you can distinguish very easily, especially if you take the precaution of pressing at the same time with the other hand on the iliac region, where the principal seat of the tumor is; and by this means, too, you can ascertain fluctuation. You can also, in this case, with advantage, join to the examination per vaginam that by the rectum also; by means of this latter mode of exploration, you are enabled to circumscribe the tumor perfectly. We have employed these different methods of examination in our patient, and we have been able to distinguish nothing in the vagina, nor in the rectum, and we concluded, therefore, that the tumor was limited to the iliac region, and that, as yet, the suppurative process was not established.

Most generally these tumors terminate by suppuration; and you see them in that case, after having run through all the phases of abundant suppuration, give rise to symptoms of purulent resorption. Fever occurs, diarrhœa follows, the cerebral phenomena manifest themselves, and the patient soon succumbs. You can, in such cases, foresee the lesions which the autopsy will reveal.

Occasionally these phlegmons are resolved; but to obtain this result you must act from the beginning with great energy, by direct and indirect antiphlogistic means, and by revulsives. You sometimes see under the influence of this treatment the pain cease, the tumor gradually diminish to the size of a small nut, and finally, after remaining so for some time, entirely disappear. When a phlegmonous tumor terminates by suppuration, the pus may diffuse itself, and take different directions. I have seen it point sometimes in the intestines, at other times in the vagina or the bladder, giving rise to a series of symptoms which alone would show the existence of a new affection. But methodical palpation made upon different parts of the abdomen, the pain which occurs when the implicated parts are pressed on, put the physician on the track of the disease he has now to deal with. When you suspect the complication I have just indicated, you should recommend the patient or the nurse to preserve the urine and the stools; you will explore, too, the vagina and rectum carefully, and you will in this manner ascertain if these organs are implicated.

From the commencement I suspected the development of the consecutive affection in question. To confirm these suspicions the urine and the fœcal matters were carefully preserved and watched, but no trace of pus was discovered in these excretions, and we were convinced that all the disease was limited to the iliac fossa. Injections of marsh mallows were thrown into the vagina, and leeches, with poultices, were applied over the seat of the tumor. The pain ceased, there was sensible diminution in size, and a little slightly elastic swelling remained, in which resolution went on slowly from day to day. The appetite returned, the strength gradually improved, and all led us to hope that all trace of the disease would in a short time disappear.

On the 9th January, having up to that period continued to do well, she was suddenly seized with severe febrile symptoms, loss of appetite, and general prostration. We suspected the occurrence of suppuration in the tumor of the iliac fossa, but we waited until the morbid phenomena became more marked. Two days after she was taken with a violent, tearing pain in the abdomen, with immediate alteration of countenance,—the tongue became dry; the lips violet; the pulse exceedingly frequent, (from 150 to

160;) the skin cold; there were repeated vomitings, which were very exhausting. She had been very restless the night preceding. In a day or two the iliac fossa became very much swollen, and pressure upon that region gave great pain; all the symptoms, in fact, of a very grave acute peritonitis were developed. Now, what had occurred to develope this peritonitis? Two causes may be suspected; either that the inflammation spread from the iliac tumor to the peritoneum, or, that there had been rupture of the cyst, and effusion of its contents into the peritoneal cavity. The first hypothesis seems to me but scarcely supposable, for a peritoneal inflammation developed in this manner would not be announced by symptoms so severe and alarming; it would have supervened more slowly, and with less violence. It is much more likely that the peritonitis in this case resulted from the effusion of fluid into the abdomen, in consequence of rupture of the iliac tumor. The prognosis was of course exceedingly grave, and we had every reason to believe that the disease would terminate speedily in death. What was to be done in the face of this new complication? Our only resource was a purely palliative treatment, which would assuage as much as possible her sufferings, remove everything which would produce any movement in the patient, augment her pains, or aggravate her condition. On this account we did not order even enemata; a little magnesia was prescribed, rather as a modifier of the gastric state than as a purgative.

On the 10th there was some improvement; the pulse fell from 150 to 120, and finally to 104. In spite, however, of this, we still regarded the condition of the patient as very desperate. She was always oppressed; an abundant, cold, clammy perspiration bathed her whole body; her pulse was small, and very compressible; her countenance was very much changed; the abdomen remained painful; the bilious vomitings continued; no alvine evacuations. The treatment now ordered consisted of mercurial frictions, iced drinks, with calomel in doses of sixteen and eighteen grains, with the object of removing the gas collected in the intestines, and to unload the rectum. Under this treatment there was decided amelioration. The flat sound, which had occupied a large portion of the abdomen, became limited to the hypogastrium. We supposed the phlegmon limited to this part; and for the purpose of assuring ourselves we made an examination per vaginam.

On introducing the finger into the vagina as far as possible, I was able to detect superiorly and posteriorly an elastic point. Believing in the existence of a purulent collection into the recto-vaginal wall, I introduced a finger into the rectum, but this exploration revealed nothing peculiar in this region. On pushing the finger as far as possible, I thought that I perceived towards the pelvis a kind of hardness, tumefaction, or œdema, which led me to suspect that the peritoneal inflammation was circumscribed to the inferior portion, had reached the pelvis, and tended to point through the intestine. In this belief the nurse was ordered to preserve the urine and stools. The next day we noticed on the surface of the matters in the vessel a thick coat of pus, of a reddish hue, and streaked with blood. Thus we had incontestable proof that the abscess had opened into the intestine. Now, how did this happen? Did the pus enter directly the intestine from the abscess, or did it do so through the medium of the peritoneum? It is difficult to decide rigorously this question; but it appears to me most probable that the pus passed from the iliac abscess into the peritoneum, and from thence into the intestine, and that there was no direct communication between the purulent collection and the intestine. It is also probable that the pus of the abscess, in reaching the peritoneum, excited suppurative inflammation in that membrane, after the recognised fact that pus produces pus; and that the pus which is now discharged from the intestine is derived in part from the abscess, but in greater part from the peritoneum itself. This is a fact very remarkable in this point of view, and one that should be noticed. At this period the hypogastrium was flat, nearly as much so as the epigastrium, whilst previously it was very salient. The intestinal convolutions, which were well defined, and prominent beneath the integuments, also disappeared; in fact, a very great change for the better occurred. But still, the patient was very far from being out of danger. The pus, although having an outlet through the intestine, could stagnate in some point; the walls of the cavity would remain open, and consequently the abscess would not be entirely emptied; and from thence all the consequences of purulent resorption and its results might be anticipated; so that, with all the actual amelioration in the patient's case, the question of the issue of the disease was very far from being settled. This state of improvement, in fact, did not last; the inflammatory symptoms reappeared in the hypogastrium, and the patient sank.

The autopsy showed us manifest traces of peritonitis; there were filamentous adhesions between the intestinal convolutions, passing from one convolution to the other, and which could be easily torn. At the inferior part of the abdomen there was a pouch containing a tumblerful of pus. We found also pus effused into the cavity of the pelvis. Evident traces of inflammation existed, too, in the broad ligament of the right side; it was in this part that the phlegmonous inflammation commenced and the abscess was formed, to discharge into the péritoneum, and from thence into the cœcum. In this part of the intestinal canal there was a perforation large enough to admit the finger, through which the purulent matter contained in the cavity of the peritoneum passed, and then passed out through the rectum. If the perforation had happened through the rectum instead of the cœcum, the chances of a favourable termination would have very much increased.

The uterus offered also evident traces of inflammation; at some points, indeed, it was gangrenous; this alteration was at a level with the primitive abscess, and is very remarkable.

Case II.—*Abscess in the left iliac fossa—Development of another similar one on the right side—Symptoms of hectic fever—Convalescence.*

At No. 5 of the same ward is a patient whose history presents a great deal of analogy to the preceding one, and who offers some interesting points relative to the subject that we are now considering. This woman, who is twenty-five years of age, entered the Hôtel Dieu about six months since. She told us that she had never had children. In the left iliac fossa there was a tumor, very appreciable on palpation. This tumor was accompanied with pains, and symptoms of febrile reaction. Twenty-two days after, the patient passed with her urine some pus, which induced us to think that the abscess had pointed into the bladder. This woman remained for some weeks in the house, where she was submitted to appropriate treatment, which principally consisted in methodical compression over that part of the abdomen which we considered the seat of the abscess, with the object of inducing adhesion of the walls of the cyst. She left the hospital on the condition of continuing the use of the bandages until the cure was complete.

Eight days since this woman was again seized with violent pain in the right iliac region, with chills, and a general state of *malaise*, to such a degree as to be obliged to return to the hospital. This time we examined her case with great attention. By touching, we discovered nothing anormal in the uterus, which retained its natural mobility. There was some sensibility, on pressure, in the iliac region, but no appreciable tumor. Some days elapsed without the occurrence of other symptoms; but we soon began to perceive traces of pus in the urine, and its existence there was confirmed by an analysis of M. Bouchardat. We concluded, then, that an abscess was developed in the right iliac fossa, similar to the one which had previously existed on the left side. On examining again *per vaginam*, we found the uterus increased in volume, without suppression of the menses, which removed all idea of pregnancy. The patient told us that she had seen, for some time past, a thick, whitish cloud in her urine, formed by a creamy substance, which we did not hesitate for an instant to recognise as pus. From all appearances the purulent collection in the left side, which had existed six months before, had never completely closed, and the pus still continued to be discharged into the bladder, as before. It is extremely rare to see iliac abscesses remain open for so long a time; still, some examples are found in the annals of our science, and I have met with some cases in my own practice. The principal phenomenon or symptom which enlightens, in these circumstances, the physician, upon the affection which he has to deal with, is the presence of pus in the urine, or in the fœcal matter.

A number of curative measures have been suggested in such cases. These means consist chiefly in the use of douches in the abscess, with the double object of cleaning out the purulent cavity, and getting rid of the pus, which might occasion subsequent grave accidents, and to dispose the cyst to heal.

Abscesses of the pelvis require, above all, the employment of compression, when the cyst persists in remaining open. All practitioners who have had much to do with this affection agree in this respect, and I myself have frequently had occasion to acknowledge the utility of the practice.

Boyer, under similar circumstances, recommended to a woman laboring under a rebellious iliac phlegmon, which would not cicatrize, to become pregnant. She did so, and was cured. You can easily understand, in this instance, how the pregnancy affected the cure; the gravid uterus exercised compression upon all the surrounding parts, thus favouring the adhesion of the sides of the abscess.

Our patient first presented an inflammatory affection, followed by a phlegmon in the left iliac region, which opened into the bladder. This patient has never been pregnant. A mild antiphlogistic treatment easily overcomes the uterine phlegmasia, but does not arrest the progress of the iliac abscess, and the patient leaves the hospital the first time alleviated, but not cured; for, for some time subsequently, as we have seen, pus continues to be found in the urine. Pain is subsequently felt in the right iliac region, and all the symptoms of the first affection appears on the opposite side. In the present case the most energetic means were employed, with, however, great fears for the result. For three weeks she continued in the same state; and, at the end of that time she was rather worse. She suffered a great deal in the hypogastric region. The urine continued to contain always the same quantity of pus, (about half an ounce,) of reddish-grey colour.

Eight or ten days subsequently the fever increased, and the volume of the uterus augmented; the hypogastrium became more painful on pressure; the general symptoms were aggravated; whilst, on the other hand, pus ceased to be discharged, until nearly every trace of it vanished. It has been remarked that in these affections, whenever the general symptoms increase, and the local pain becomes more intense, the quantity of pus diminishes, and *vice versa;* on the reëstablishment of the purulent discharge the accidental phenomena cease, and the patient returns again to the same state as before. De Halle first remarked this coincidence. This aggravation of the general and local symptoms led us to suspect a retention of pus in the abscess. But the pus in a short time reappeared; and this time, in place of being discharged into the bladder, and appearing in the urine, it was discharged through the rectum. In the vessel, and in the matters contained in it, there was an ounce of pure pus, mixed with the solid fœcal matter. Nearly immediately after the reappearance of the pus the pulse fell to about seventy or seventy-five, the hypogastric pain diminished, and the patient demanded something to eat. We endeavoured to discover the point at which the pus reached the rectum. On examination *per vaginam*, I detected a doughiness, indicating a tumor, at some distance. The rectal examination, by means of which much more light could have been thrown on the nature of the local affection, was rendered almost impracticable by a fissure which existed at the margin of the anus.

After some days, however, on proceeding with great caution, we were enabled to introduce the finger a sufficient distance to explore the whole region, but were unable, in spite of all the care used, to distinguish the spot in the rectum at which the pus penetrated.

Enemata were ordered, which had the double advantage of washing out the abscess and maintaining the intestine free.

On the 7th January there was decided amendment; no more pus was discharged by the rectum, although the urine continued to be somewhat troubled. The same treatment was continued, with much methodical compression. From this time convalescence proceeded rapidly.

There was, however, towards the latter part of the month, a return of the febrile symptoms, with abundant nocturnal sweats, which occurred principally about daybreak. In spite of this the appetite continued good. These phenomena, which so frequently occur in bed-ridden patients, are often referrible to the existence of tubercles. It is that, at least, which you most commonly see in hectic fever without any new appreciable cause. It must be acknowledged, however, that hectic is not always the result of tubercularization; it may be the effect of extensive suppuration, which is certainly its cause in the present instance. Our patient has had extensive and deep suppuration, which was discharged both into the bladder and the rectum; the pus then ceased to be discharged by these artificial means. Now, it is possible that there is some pus remaining in the abscess, which causes the hectic fever. Deep seated abscesses of the abdomen often occasion this febrile state; and the prudent physician, before proceeding to pronounce upon the existence of tubercles, will duly consider all the circumstances which can explain the existence and persistence of hectic.

In this state the patient continues. She passes occasionally a few drops of pus in her urine; the diarrhœa is arrested. Finding that her prolonged stay in the hospital would have an unhappy influence upon her *morale*, we shall give her a discharge, recommending her to be particular in her diet, and to let it be as nutritious as possible. It may be hoped that, as she gains flesh, the sides of the abscess will adhere, and that a radical cure will take place. Without this condition, on the contrary, a relapse will be likely to occur.

CASE III.—*Iliac phlegmon terminating by resolution.*

At No. 38 of the same ward, in the next bed to this patient, is another woman, about thirty years of age, who entered the service on the 28th of October. On her entrance she had erysipelas of the face, and at the same time she complained of violent pain in the hypogastric region, principally in the right side. After suitable treatment the erysipelas disappeared, but the fever continued, as well as the local abdominal symptoms. On exploring the abdomen carefully we distinguished an oblong tumor, moveable under the finger, and sensible on pressure. It was a phlegmon in the right iliac region. A vaginal exploration revealed nothing to us in that quarter. The treatment was nearly the same as in the other case. After having exhausted the antiphlogistics, (bleedings, baths, cataplasms,) we recurred to resolutives, to mercurial frictions, and by these means succeeded in reducing the tumor about one-half, and rendering it less hard and less sensible to pressure. In the mean time the general and local symptoms disappeared; her diet was improved; her appetite and digestion are good; and there is every reason to believe that her recovery will be definitive.

